# [Retracted] Improved ataxia telangiectasia mutated kinase inhibitor KU60019 provides a promising treatment strategy for non-invasive breast cancer

**DOI:** 10.3892/ol.2026.15605

**Published:** 2026-04-20

**Authors:** Ying Zhu, Changfei Mao, Jianzhong Wu, Shuchun Li, Rong Ma, Haixia Cao, Minghua Ji, Changwen Jing, Jinhai Tang

Oncol Lett 8: 2043–2048, 2014; DOI: 10.3892/ol.2014.2444

Subsequently to the publication of the above article, a concerned reader drew to the Editor's attention that, regarding the migration and invasion assay data shown in Fig. 3A on p. 2046, of the ten data panels, the Migration/Dox and Migration/Dox&KU60019 data panels contained an overlapping section, while the Invasion/Blank, Invasion/DMSO and Migration/KU60019 data panels also contained overlapping data, such that this trio of panels were apparently derived from the same original source. In addition, in Fig. 4E, the E-cadherin blots featured in the first and the second lanes in the gel slice appeared to be strikingly similar, suggesting that this figure may have potentially undergone inappropriate editing.

After having evaluated these data independently in the Editorial Office, the concerns of the reader were shown to have been well-founded. Therefore, in view of the uncertainties regarding the assembly of some of the western blot data in Fig. 4E, and given the number of overlapping data panels that were identified in Fig. 3A, the Editor of *Oncology Letters* has decided that this paper should be retracted from the Journal on account of a lack of confidence in the presented data. The authors were asked for an explanation to account for these concerns, but the Editorial Office did not receive a reply. The Editor apologizes to the readership for any inconvenience caused.

